# Comparative Evaluation of Solubility of Gutta-Percha in Three Different Solvents: A Cone-Beam Computed Tomography (CBCT) Study

**DOI:** 10.7759/cureus.26788

**Published:** 2022-07-12

**Authors:** Yesh Sharma, Sharath P Kumar, Girish S A, Divya Pandey, Mujahid Ahmed, Shadab Ahmed

**Affiliations:** 1 Department of Conservative Dentistry and Endodontics, Pacific Dental College and Hospital, Udaipur, IND; 2 Department of Conservative Dentistry and Endodontics, Sri Siddhartha Dental College and Hospital, Tumkur, IND; 3 Department of Conservative Dentistry and Endodontics, Chandra Dental College, Lucknow, IND

**Keywords:** cbct, chloroform, xylene, orange oil, gutta percha

## Abstract

Background

Gutta-percha is the most frequently used filling material for root canal obturation. This thermoplastic material fulfills the primary requisites for root canal filling, one of which is easily removable material in cases of endodontic retreatment. The most commonly used solvents were chloroform, xylene, and orange oil due to their effectiveness in dissolving and removing maximum gutta-percha in a minimum time.

Aims

The aim is to evaluate and compare the solubility of gutta-percha in three different organic solvents, i.e., orange oil, xylene, and chloroform.

Methods and material

Forty extracted mandibular second premolars with a single canal were selected. The sample was categorized into control, orange oil, xylene, and chloroform. Access cavity preparations with cleaning and shaping were performed by k files and rotary files, followed by obturation. Two drops of assigned solvent were placed on the orifices of the obturated canal, and corona-gutta-percha ha was removed by gates glidden drills. Cone-beam computed tomography (CBCT) images were taken before and after the gutta-percha removal, and the solvents' efficacy was assessed.

Statistical analysis

The statistical test applied for the analysis was one-way analysis of variance (ANOVA). The confidence interval and p-value were set for this test at 95% and < 0.05, respectively.

Results

Gutta-percha Removal was found to be maximum with orange oil (Group II) followed by xylene (Group III) >Chloroform (Group IV) >Control Group. The Statistical Analysis by ANOVA revealed a significant difference between the four groups with a p-value of less than 0.05. Furthermore, the pair-wise comparison revealed that the amount of gutta-percha removal with orange oil significantly differs from xylene and chloroform, with a significance level of less than 0.021,001. However, there was no difference observed between control and chloroform.

Conclusions

Within the limitations of this in vitro study, it can be concluded that the maximum amount of gutta-percha removal was found in the orange oil group. The amount of gutta-percha removal with orange oil significantly differs from xylene and chloroform, with a significance level of less than 0.02,001. Hence there was no difference observed between Control and Chloroform groups.

## Introduction

One of the keys to pulp space therapy's success is to obturate the prepared root canal space adequately. Furthermore, it seals the complex root canal system from the periodontal ligament and bone, ensuring the attachment apparatus's health against the breakdown of Endodontic origin [1]. The 3d Hermetic Seal was achieved by proper obturation of the prepared root canal space to prevent the recurrence of bacterial infection [2,3]. Gutta-percha is the most frequently used filling material for root canal obturation. This thermoplastic material fulfills the primary requisites for root canal filling, one of which is easily removable material in cases of endodontic retreatment. The methods used to perform retreatment are mechanical, thermal, chemical, or even associated, and special instruments such as ultrasound can be used [4,5]. Successful removal of gutta-percha and sealer is an essential step during retreatment; therefore, removing the maximum amount of filling material from inadequately prepared and filled root canal systems appears to be essential to uncovering remaining necrotic tissue or bacteria that may be responsible for persistent disease. Most current methods for gutta-percha removal in practice include using hand files, Gates Glidden drills, and rotary files with or without solvents. The most commonly used solvents were chloroform, xylene, and orange oil, being the gold standard due to their effectiveness in dissolving and removing maximum gutta-percha in a minimum time has been widely used. This study aimed to compare the effectiveness of xylene, orange oil, and chloroform in gutta-percha removal during endodontic retreatment on human extracted permanent teeth.

## Materials and methods

Subjects and methods

This in-vitro study was conducted in the Department of Conservative Dentistry and Endodontics, Maharaja Ganga Singh Dental College and Research Centre Sri-Ganganagar, Rajasthan, and ORA Cone-Beam Computed Tomography (CBCT) and Scan Hub Jaipur, Rajasthan. 

Methodology

The present study was conducted in-vitro on freshly extracted 40 human permanent mandibular second premolars collected from the Department of Oral and Maxillofacial Surgery, Maharaja Ganga Singh Dental College and Research Centre, Sri Ganganagar, Rajasthan. The criterion for selection of teeth was non-carious and intact mandibular second premolars. After extraction, the teeth were cleaned of blood and debris in running water, followed by soaking them in 5.25% sodium hypochlorite for 24 hours. The remaining periodontal tissue and calculus were removed with a scaler.

The teeth were then stored in normal saline at room temperature till the study was conducted. The teeth were then examined under a dental operating microscope to rule out any teeth with pre-existing root fractures. Access preparation was done using round No.2 bur attached to a high-speed contra-angle air- rotor hand-piece. The gross pulp tissue was removed using a barbed broach and a no. 15 K file was introduced into the canal until it was visually observed exiting through the apical foramen. Working length was established by subtracting 0.5 mm from this length.

All canals were prepared mechanically with Rotary Protaper files using a crown-down technique in the sequence of shaping instruments X1, X2, till X3 up to the working length. During the preparation, all teeth were irrigated alternatively with 10 ml of 5.25% sodium hypochlorite and 10 mL of 17% EDTA.

Prepared specimens were irrigated with normal saline. The canals were then obturated with gutta-percha cones and using Seal apex as a root canal sealer. After obturation, CBCT images of all groups were taken. The 40 teeth were then randomly divided into four experimental groups of 10 teeth each as follows (Table 1).

**Table 1:** Distribution of the teeth from all groups.

Groups	Sample code	Sample Size
Total number of prepared specimens		40
Group I (Control)	Group I	10
Group II (Orange oil)	Group II	10
Group III (Xylene)	Group III	10
Group IV (Chloroform)	Group IV	10

Group I - control group

**Figure 1:**
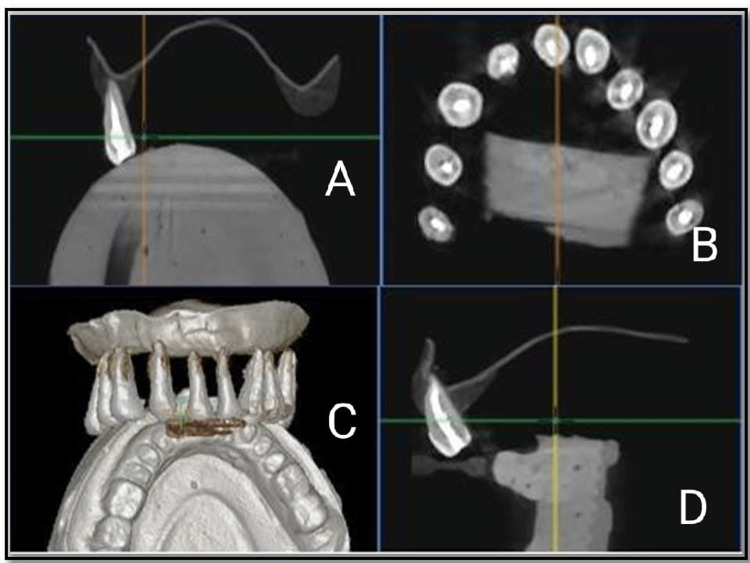
Cone-beam computed tomography images of Control group before gutta-percha removal. (A, D) Mid sagittal view of tooth, (B) coronal view, and (C) complete view

In this group, Biomechanical preparation was done till X3 Rotary Protaper files. Sealer was delivered to the canal with the help of lentulospiral. The canals were then obturated with gutta-percha cones and using Sealapex as a root canal sealer, and postoperative CBCT images were taken after that (Figure 1).

**Figure 2:**
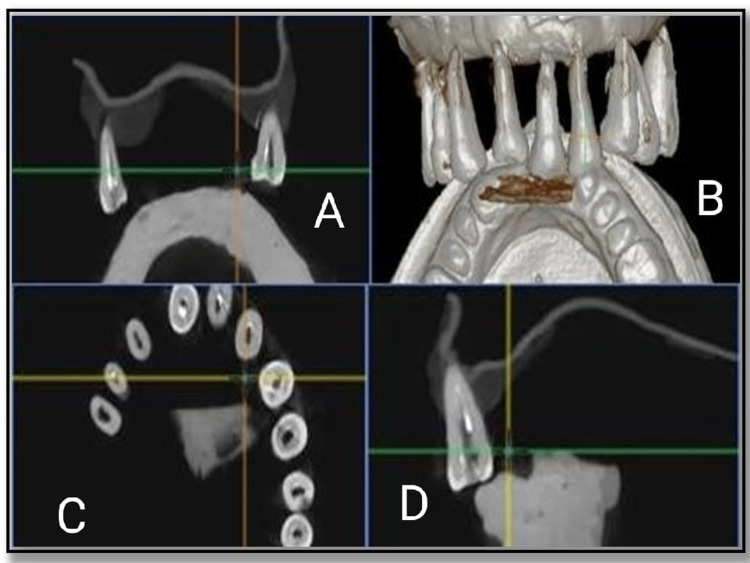
Cone-beam computed tomography images of Control group after gutta-percha removal. (A, D) Mid sagittal view of tooth, (B) complete view, and (C) coronal view

Gates Glidden drills #1, 2, and 3 were used to remove coronal gutta-percha. The canal was re-instrumented using H-files in a circumferential quarter-turn push-pull filing motion for removal of remaining gutta-percha. The teeth were then stored in distilled water for four weeks. CBCT images of the Control group after gutta-percha removal was taken (Figure 2).

Group II - orange oil

**Figure 3:**
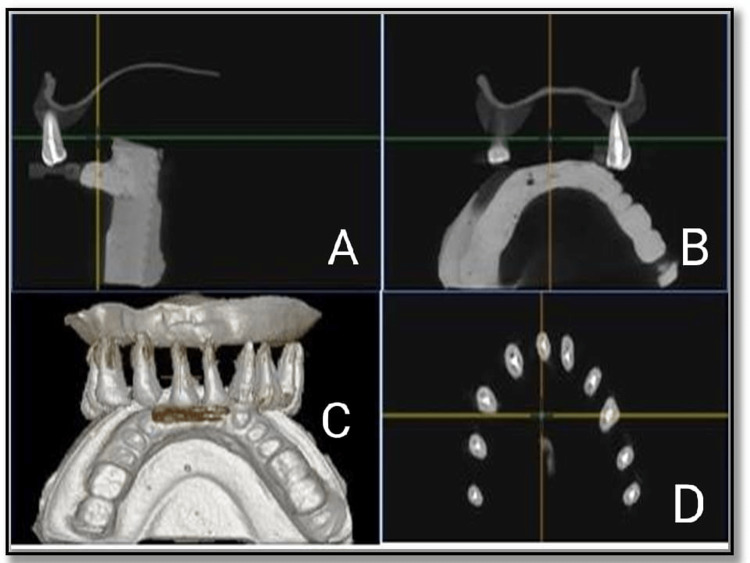
Post obturation CBCT images - orange oil group. (A, B) Mid sagittal view of tooth, (C) complete view, and (D) coronal view CBCT - Cone-Beam Computed Tomography

In this group, biomechanical preparation was done till X3 Rotary Protaper files. Sealer was delivered to the canal with the help of lentulospiral. The canals were then obturated with gutta-percha cones and using Sealapex as a root canal sealer, and postoperative CBCT images were taken after that (Figure 3).

**Figure 4:**
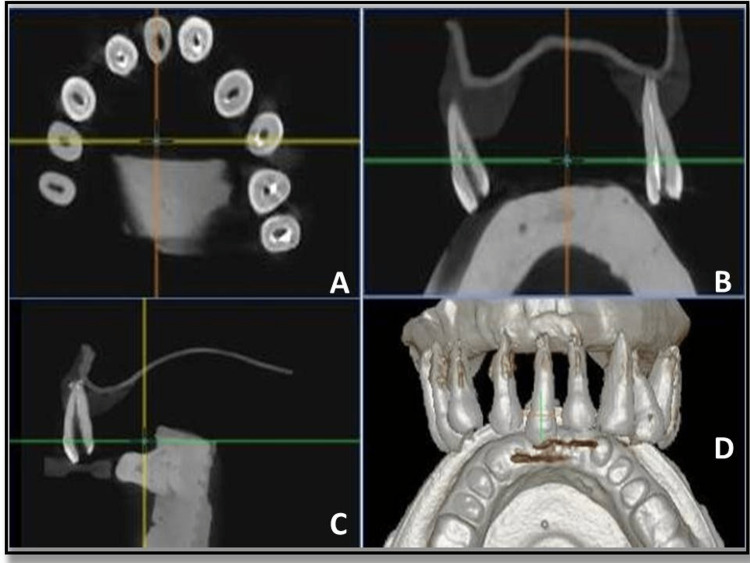
Cone-beam computed tomography images of orange oil group after gutta percha removal. (A) Coronal view, (B, C) mid sagittal view of tooth, and (D) complete view

The teeth were then stored in distilled water for four weeks. Finally, two drops of orange oil were placed on the orifice of the obturated canal. Gates Glidden drills #1, 2, and 3 were used to remove coronal gutta-percha. The canal was re-instrumented using H-files in a circumferential quarter-turn push-pull filing motion for removal of remaining gutta-percha. CBCT images of the orange oil group after gutta-percha removal was taken (Figure 4).

Group III - xylene

**Figure 5:**
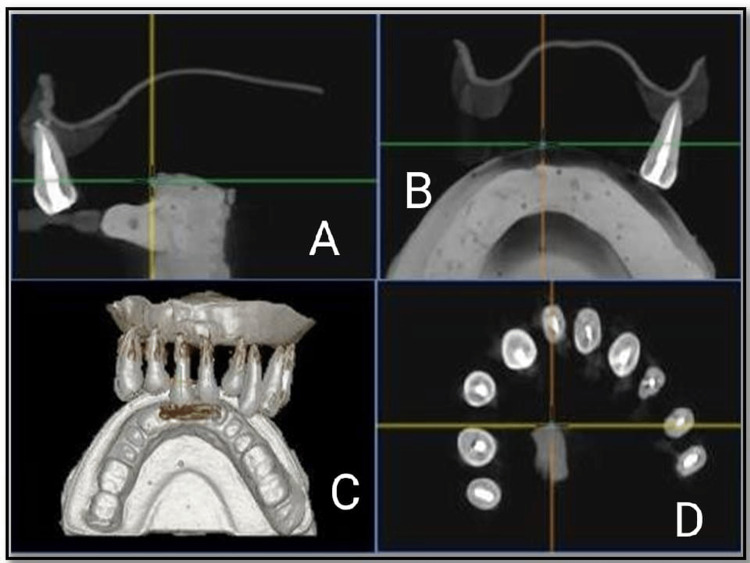
Post obturation CBCT images - xylene group. (A, B) Mid sagittal view of tooth, (C) complete view, and (D) coronal view CBCT - Cone-Beam Computed Tomography

In this group, biomechanical preparation was done till X3 Rotary Protaper files. Sealer was delivered to the canal with the help of lentulospiral. The canals were then obturated with gutta-percha cones and using Sealapex as a root canal sealer, and postoperative CBCT images were taken (Figure 5).

Group IV - chloroform

**Figure 6:**
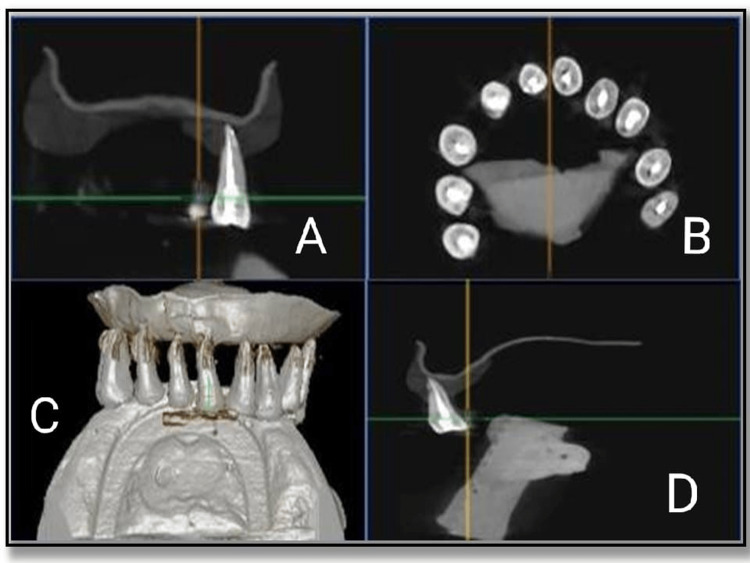
Post obturation CBCT images - chloroform group. (A, D) Mid sagittal view of tooth, (B) coronal view, and (C) complete view CBCT - Cone-Beam Computed Tomography

In this group, biomechanical preparation was done till X3 Rotary Protaper files. Sealer was delivered to the canal with the help of lentulospiral. The canals were then obturated with gutta-percha cones using Sealapex as a root canal sealer, and postoperative CBCT images were taken after that (Figure 6).

After completely removing of gutta-percha using a different organic solvent, the teeth from all groups were examined and recorded were canal curvature (in degrees), length of canal obturated (mm), length of remaining gutta-percha (mm), location of remaining gutta-percha (coronal apical or middle third of the canal), and grading according to Hulsman and Slotz Scale.

Statistical analysis

The recorded data were compiled in a spreads sheet computer program (Microsoft Excel 2010) and then exported to data of SPSS version 19 ( SPSS Inc., Chicago, IL, U.S.A.). Descriptive statistics included computation of mean and standard deviation. The statistical test applied for the analysis was a one-way analysis of variance (ANOVA). The confidence interval and p-value were set for this test at 95% and < 0.05, respectively.

## Results

The present study evaluated the complete removal of gutta-percha from root canal using different solvents in freshly extracted 40 Human permanent mandibular second premolars. The teeth from all groups were examined, and the location of the remaining gutta-percha (coronal apical or middle third of the canal) was graded according to Hulsman and Slotz Scale. 

Length of remaining gutta-percha (Hulsman and Slotz Scale)

The length of the remaining gutta-percha was assessed according to the Hulsman and Slotz scale:

Class I: No root canal filling material.

Class II: One to three small isles (<2mm long) of root canal filling material. 

Class III: More than three small isles (<2mm long) of root canal filling material. 

Class IV: One large piece (>2mm long) of root canal filling material.

Class V: Root canal filling material > 5mm long.

Class VI: Several isles of root canal filling material (>2mm long)

The intra-group comparison showed that Gutta-percha removal was found to be maximum with orange oil (Group II) followed by xylene (Group III) >Chloroform (Group IV) >Control Group (Table 2).

**Table 2:** The length of residual gutta-percha.

SAMPLE NO.	GROUP-I (CONTROL)	GROUP-II (ORANGE OIL)	GROUP-III (XYLENE)	GROUP-IV (CHLOROFORM)
1	Class III	Class II	Class II	Class III
2	Class II	Class I	Class II	Class III
3	Class IV	Class II	Class II	Class II
4	Class IV	Class II	Class II	Class III
5	Class III	Class II	Class II	Class II
6	Class III	Class II	Class II	Class I
7	Class III	Class II	Class I	Class IV
8	Class II	Class I	Class I	Class III
9	Class III	Class II	Class II	Class II
10	Class IV	Class II	Class II	Class II

The statistical analysis by ANOVA revealed a significant difference between the four groups with a p-value less than 0.05. The pair-wise comparison revealed that the amount of gutta-percha removal with orange oil significantly differs from xylene and chloroform, with a significance level of less than 0.021,001. However, there was no difference observed between Control and Chloroform (Table 3).

**Table 3:** Intra-group comparison of the remnant of gutta-percha.

Groups	p value	Significance level
Group I vs II	0.005	Significant
Group I vs III	0.041	Significant
Group I vs IV	0.838	Not Significant
Group II vs III	0.021	Significant
Group II vs IV	0.001	Significant
Group III vs IV	0.004	Significant

## Discussion

The main aim of retreatment is to regain access to the apical foramen by completely removing the root canal filling material. Removing the most filling material from inadequately prepared or filled root canal systems appears to be essential to uncovering remaining necrotic tissue or microorganisms responsible for the persistent disease and enabling thorough chemomechanical disinfection and reinstrumentation of the root canal system to promote the healing of periradicular tissue [6,7]. Moreover, the residual filling material may interfere with the adhesion and adaptation of root filling in subsequent treatment, which may affect the success rate. With reference to the standardization protocol proposed by Al-Omari and Dummer in 1995, in this study, the crowns of the teeth were removed, and the length was standardized at 16mm to allow better visualization of root canal morphology and to eliminate any coronal interferences during canal preparation and retreatment [8]. The amount of filling material remaining inside the canal after the retreatment procedure was assessed radiographically, or the roots were split longitudinally. The residual gutta-percha and sealer were measured linearly or using a scoring system. In addition, computed tomography has been used in endodontics.

The most crucial advantage of CBCT in endodontics is that it demonstrates anatomic features in three dimensions that intraoral and panoramic images cannot. Therefore, it can be Safely Concluded that the maximum amount of gutta-percha removal was found to be with orange oil (Group II) followed by xylene (Group III) >Chloroform (Group IV) >Control Group (Group I).

The results of this study are in accordance with that by Kulkarni et al. who evaluated and compared the effectiveness of eucalyptus oil, orange oil, and clove oil in dissolving resin‐coated gutta‐percha (RCGP) cones [9]. The results of the present study are similar to the study conducted by Subbiya et al. [10]. They evaluated the antibacterial efficacy to assess the minimum inhibitory concentration (M.I.C.) of three gutta-percha solvents - R.C. Solve (orange oil), Endosolv-E, and xylene against E. faecalis. The results of this present study are not in accordance with the study conducted by Alberto Dagna et al. [10], who evaluated the effectiveness of different alternative solvents with the ability to dissolve gutta-percha that can be regarded as a practicable alternative to chloroform. They concluded that chloroform is the most effective solvent. Other solvents, such as G.P.R. and orange solvent, could be considered an alternative to chloroform, even if they are less efficient.

## Conclusions

The present study evaluated and compared the solubility of gutta-percha in three organic solvents used in endodontics in 40 mandibular second Premolars. Access opening of all samples and instrumentation was done using K file and rotary Protaper files till X3. Later the teeth were allocated to four different experimental groups. A standardized amount of root canal sealer, i.e., Sealapex, was applied via lentulo spiral, and obturation of samples was done. Two drops of the assigned solvent were placed on the orifice of the obturated canal, gates glidden drills were used to remove coronal gutta-percha, and the canals were re-instrumented using H files. CBCT images of the Control group after gutta-percha removal were taken.

This study found the maximum amount of gutta-percha removal using all groups of solvents. It was observed that gutta-percha removal was found to be maximum with orange oil (Group II) followed by xylene (Group III) >Chloroform (Group IV) >Control Group. The statistical analysis by ANOVA revealed a significant difference between the four groups with a p-value less than 0.05. The pair-wise comparison revealed that the amount of gutta-percha removal with orange oil significantly differs from xylene and chloroform, with a significance level of less than 0.021,001. However, there was no difference observed between Control and Chloroform groups. Within the limitations of this in vitro study, it can be concluded that the maximum amount of gutta-percha removal was found in the orange oil group. The amount of gutta-percha removal with orange oil significantly differs from xylene and chloroform, with a significance level of less than 0.02,001. Hence, there was no difference observed between Control and Chloroform groups.
